# Generating the perception of choice: the remarkable malleability of option‐listing

**DOI:** 10.1111/1467-9566.12766

**Published:** 2018-08-03

**Authors:** Merran Toerien, Markus Reuber, Rebecca Shaw, Roderick Duncan

**Affiliations:** ^1^ Department of Sociology University of York York UK; ^2^ Academic Neurology Unit University of Sheffield Sheffield UK; ^3^ Social Sciences Division University of Oxford Oxford UK; ^4^ Department of Neurology Christchurch Hospital Christchurch New Zealand

**Keywords:** Conversation analysis, doctor‐patient interaction, patient choice, option‐listing, neurology

## Abstract

The normative view that patients should be offered more choice both within and beyond the UK's National Health Service (NHS) has been increasingly endorsed. However, there is very little research on whether – and how – this is enacted in practice. Based on 223 recordings of neurology outpatient consultations and participants’ subsequent self‐reports, this article shows that ‘option‐listing’ is a key practice for generating the perception of choice. The evidence is two‐fold: first, we show that neurologists and patients overwhelmingly reported that choice was offered in those consultations where option‐listing was used; second, we demonstrate how option‐listing can be seen, in the interaction itself, to create a moment of choice for the patient. Surprisingly, however, we found that even when the patient resisted making the choice or the neurologist adapted the practice of option‐listing in ways that sought acceptance of the neurologist's own recommendation, participants still agreed that a choice had been offered. There was only one exception: despite the use of option‐listing, the patient reported having no choice, whereas the neurologist reported having offered a choice. We explore this deviant case in order to shed light on the limits of option‐listing as a mechanism for generating the perception of choice.

## Introduction

The view that patients should be offered more choice within publicly‐funded healthcare settings has been increasingly endorsed over the last two decades (Dent 2006). Indeed, Costa‐Font and Zigante (2016: 409) describe the expansion of the ‘choice agenda’ as ‘a dominant reform’ taking place across many European National Health Service (NHS) systems, ‘where choice has traditionally been limited, relative to Social Health Insurance (SHI) systems where choice is traditionally institutionalised’. More broadly, the notion of patient choice connects (albeit in complex ways, as we recognise in our Discussion, below) with the widespread view that some version of ‘shared decision‐making’ is the ideal approach to health care in the 21st century (e.g. Elwyn and Edwards 2009). Policy documents relating to the United Kingdom's NHS – the setting for the study reported here – endorse both the general principles of shared decision‐making and a commitment to patient choice, specifically. This is exemplified in the opening of the NHS Choice Framework (2016), which moves quite fluidly between the notion of ‘choice’ and wider ideals such as ‘patient empowerment’. Similarly, the Department of Health (2007: 6) document, *Choice Matters*, lays out a broad vision for the implementation of patient choice within the NHS, and specifies forms of choice to be made available (e.g. which hospital to attend when referred by a GP) within a target timescale. Patient choice in the UK's NHS is, then, both carefully delimited and articulated as a broad aspiration.

Despite this, there has been little attempt to address the question: what constitutes ‘patient choice’ in practice? While patients’ legal rights to choose may be (relatively) easy to pin down, the enactment of choice – during face‐to‐face decision‐making – is far from self‐evident. The few studies that have investigated choice in interaction have shown significant discrepancies between policy and practice. Pilnick (2008) notes that the UK's NICE (National Institute for Health and Care Excellence) guidelines give pregnant women the right to choose whether to undergo screening for Down syndrome. Accordingly, she found that midwives routinely articulated this right to women. Nevertheless, they tended to present the decision such that it became more a matter of ‘assent’ than of independent choice. Investigating how care home staff enact the requirement to offer choice to people with intellectual difficulties, Antaki *et al*. (2008) similarly found that – despite staff orientations to residents’ right to choose – the decision‐making practices employed often worked against the resident making a choice.

Patients report an important distinction between having a choice and making one, and between the appearance of choice and substantive choice (Barnett *et al*. 2008). Crucially, while patients – in a study of NHS primary care in the UK – valued having a (substantive) choice, they often reported not wanting to make the decision. Similarly, Ford *et al*. (2003) found that participants (lay people, academics and NHS practitioners) believed choice should include the patient's right to decide how much involvement in decision‐making they want. Such studies indicate a multi‐dimensional understanding of choice and urge caution in assuming that it will always be desirable from the patient's perspective. As Pilnick (2008: 512) has argued:

What the offering and exercising of choice actually looks like in practice […] remains unclear. The potential implications of these interactional processes, though, are immense, since […] ‘good’ practice is ultimately achieved through interaction rather than through policy or regulation.

The present article takes up the challenge in this quote, seeking to develop our understanding of how choice is enacted. We provide an analysis of decision‐making sequences in neurology consultations, and consider these in light of patients’ and neurologists’ reports of whether a choice was offered. We show that option‐listing – a practice identified in our pilot study (Toerien *et al*. 2013) – was strongly associated with the reported perception that choice had been offered. This is not surprising given that option‐listing, being akin to providing a menu, is a widespread mechanism for offering choice across a range of institutions: from restaurants to hair salons, car washes to telephone banking. More surprising, we argue, is our finding that the association between option‐listing and the perception of choice remained even in instances where patient choice was not – or, at least, not unambiguously – being enacted in practice. We refer to this as the ‘malleability’ of option‐listing because the practice may be adapted, and responded to, in highly variable ways, including the following:

- option‐listing may serve to offer a choice, but this may not be taken up by the patient;
- option‐listing may serve to offer a choice, but this may be followed immediately by a recommendation for one option; and
- the machinery of option‐listing may be used, but – in contrast with its use to lay out alternatives from which the patient might choose – it can serve to pursue acceptance of the neurologist's recommended option.

**Figure 1:**
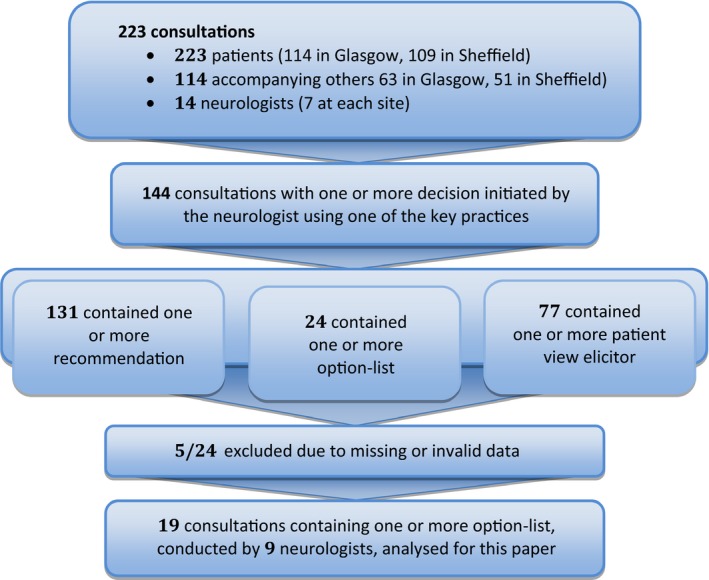
Number of recordings collected overall, key practices analysed for wider project, and consultations included in the analyses reported here

We explore this ‘malleability’ of option‐listing, showing that neurologists and patients still agreed that choice had been offered even when the patient did not actually make the choice or when the neurologist sought acceptance of their own preferred option. Only in one consultation containing option‐listing (out of 19 for which we have valid self‐report data, see Figure 1) did patient and neurologist not agree that choice had been offered. We explore this deviant case in order to shed light on the limits of option‐listing as a mechanism for generating the perception of choice.

## Methods

### Study context

This article reports findings from a study of patient choice in neurology, funded by the UK's National Institute for Health Research (see Reuber *et al*. 2015). Data were collected in the outpatient departments of two neuroscience centres, in Glasgow and Sheffield. Neurology was chosen because patients’ involvement in decisions about their care is considered important in chronic diseases generally, and certain neurological conditions specifically (Department of Health 2005). This is the case across a wide range of national settings (see Pietrolongo *et al*. 2013). Yet there is evidence that neurology patients may experience decision‐making as clinician‐dominated (McCorry *et al*. 2004) and that efforts to involve patients may not always be ‘a prominent part of patient care’ (Pietrolongo *et al*. 2013: 6). This makes neurology an important site for investigating how the patient choice agenda is being implemented.

### Data collection

Our main data source was audio and video‐recordings of consultations, collected in 2012. Participants also completed questionnaires after their consultation, which included the questions: ‘Did you give the patient a choice about treatment or further management?’ and ‘Did the doctor give you a choice about any tests or treatment you might have or the next step in the management of your condition?’ Patients were sent an information sheet at least 48 hours prior to their appointment. On arrival at the clinic they were invited to discuss the study with a research assistant, who took written consent. In total, 66 per cent of those approached agreed to take part. Participants could opt for audio or video‐recording. Ethical approval was granted by the NRES Committee for Yorkshire & the Humber (South Yorkshire) on 11 October 2011.

### Data analysis

Our primary approach was conversation analysis (CA). CA is a micro‐analytic, systematic methodology, widely recognised as the leading approach for investigating how doctor‐patient interaction functions (Heritage and Maynard 2006). CA understands talk not simply as information transfer, but as a way of performing social actions. Since any action may be performed in different ways, analysis focuses on how something gets done, and the interactional consequences thereof. In our wider study, we identified all instances of decision‐making about current treatments, investigations and referrals, and examined how the decision‐making process was initiated, pursued and concluded. We found three key practices that neurologists used to initiate and pursue decisions: recommendations (e.g. you should try x), option‐lists (e.g. you could try x or you could try y), and patient view elicitors (e.g. what do you want to do? How do you feel about trying x?) (Reuber *et al*. 2015). This article reports our analysis of the 19 consultations that contained at least one instance of option‐listing, for which we have valid self‐report data regarding the neurologists’ and patients’ perception of whether the neurologist offered the patient a choice (see Figure 1).

To compare participants’ reports of choice with what took place in the interactions, we divided the recordings into four subsets, discussed below (Table 1). We sought patterns across these with respect to how the decision‐making was accomplished. The aim was to discover, inductively, what might have been hearable in the consultations as ‘choice’. Our discovery that option‐listing was associated almost exclusively with the agreement that a choice had been offered led to the more detailed analyses that we report here.

**Table 1:** Agreement and disagreement about presence of choice

Total n = 196	Neurologist: Choice ‐ Yes	Neurologist: Choice ‐ No
Patient: Choice ‐ Yes	Subset 1. Agree (53.6%, n = 105)	Subset 2. Disagree (17.9%, n = 35)
Patient: Choice ‐ No	Subset 3. Disagree (14.3%, n = 28)	Subset 4. Agree (14.3%, n = 28)

## Findings

### The perception of choice

Overall, there was a fairly high level of perceived choice on offer in our dataset: in 67.9 per cent (146/215) of cases for which we have the relevant questionnaire data, the neurologist reported offering the patient a choice, and in 71.8 per cent (145/202) of cases for which we have the data, the patient reported being offered a choice. Table 1 shows the nature of this agreement across the four possible permutations for the 196 consultations for which data were available from both neurologist and patient. As the table shows, in just over half of these cases, there was agreement that a choice had been offered. See Wiseman *et al*. (2016) for a statistical exploration of this dataset.

Our qualitative analysis revealed a striking finding: with just one exception (i.e. 1/19, see Figure 1), option‐listing was only present in those consultations (for which we have valid data) where neurologist and patient agreed a choice had been offered (Table 1, subset 1). This leaves numerous consultations in the ‘agree choice’ subset in which decision‐making was handled in another way (e.g. using forms of recommendation or patient view elicitor). However, the finding of such a clear association between option‐listing and self‐reported perception of choice was reason enough to examine the practice further. When considered in light of how option‐listing was enacted, this pattern became even more remarkable, as we show across the remaining analytic sections.

### Having vs. making a choice

As demonstrated previously (Reuber *et al*. 2015), option‐listing consists, in its full form, of three components. Component 1 entails some indication by the neurologist that there is a decision yet to be made. Component 2 entails the subsequent formulation of a list of options, sometimes labelled as such (e.g. ‘option one is … option two is … option *n* is …’), and sometimes embedded within an interrogative (e.g. ‘would you like to x … would you like to y … would you like to z …’), some of which were either/or choices. Doing nothing was sometimes included explicitly. Component 3 entails inviting the patient to announce their views on the options and/or to select one. We refer to this component as a patient view elicitor (PVE), encompassing turns that ask what the patient wants, how they feel, and what they think about the options.

The three‐component package is illustrated in Extract 1 (see boldface across lines 1–116). Having already advised changing one of the patient's anti‐epileptic drugs, the neurologist sets up a decision regarding what to take instead. At lines 1–2 he projects a forthcoming list (component 1). At lines 7, 40 and 72, he names three options (component 2). At lines 115–116, he invites the patient's view on the options (component 3), which the patient provides (see boldface lines 117–118, 127, 151–152, 156, 180). For a key to the transcription conventions used in CA, please see: http://www.paultenhave.nl/Transcription-DCA-2.htm. The identifiers show where the consultation was recorded (Glasgow or Sheffield), the number of the recording (numbered consecutively at each site from 001), followed by a two‐digit number for each clinician.

Extract 1

**Table:** G10905: Epilepsy

01	Neu:	.hhh U:m:: (1.0) **possibilities. (0.9) .tch Okay let me**
02		**talk through these.**
03		(0.2)
04	Pat:	[Yeah.
05	Neu:	[.hhh E:r (2.0) **let's start with the oldest one first.**
06		(0.3)
07	Neu:	**Er carbamazepine. (0.2) Tegretol.** (.) .hhh drug (0.2)
08		good drug, (0.4) still useful against localisation
09		related epilepsy, .hh e::r invented, roughly ‘60s ‘70s,
(Neurologist describes pros and cons of the drug)		
39	Neu:	.tchhhh **O** **ther (0.1) possibilities. (0.2) E::r another**
40		**drug call:ed u::m (.) vi** **gab** **atrin, (0.2) Sabril,** (0.5)
41		slightly newer drug,
42	Pat:	Mh[m,
43	Neu:	[brought in in the late 1980s, .hhh good drug (0.3)
(Neurologist describes pros and cons of the drug)		
70	Neu:	.hhh Um **so that's Tegretol (1.0) the vigabatrin, (0.2)**
71		**and the last one to talk about is a drug called**
72		**retigabine.** (.) .hhh u::m which is a new drug, (0.7)
73		given a licence: (0.7) e:::r about (.) nine months ago,
74		(0.2)
75	Neu:	.hh e::r I've used it (0.3) a handful of times.
76		(0.2)
77	Neu:	.hhhh Er, quite early days yet. It works in a completely
78		different way from other antiepileptic drugs…
(Neurologist describes pros and cons of the drug)		
115	Neu:	.hhhhh hh. **Of those three:::, (0.6) do you**
116		**have any 0.2) thoughts=do you have any: hhh. (0.5)**
117	Pat:	**We::ll I'm not too keen on the:: (0.2)** **se** **cond one that**
118		**you mentioned,**
119	Neu:	Mm:.
120		(0.3)
121	Pat:	U::m (0.1)
122	Neu:	>With the< vision,
123		(0.2)
124	Pat:	Mhm::,
125		(0.7)
126	Neu:	[Okay
127	Pat:	**[Yeah that does kinda:: (0.3) [put me off.**
128	Neu:	[I know
129		(0.1)
130	Neu:	and the weight gain.
131	Pat:	Yeah.
132	Neu:	I've not‐ [to be honest I've not [done a great job of
133	Pat:	[Yea::h [that's one as well.
134	Neu:	£selling it but it‐£ you kno:w…
(Neurologist explains how this drug, while not being the most straightforward option, is on “the shortlist”)		
151	Pat:	**The‐ the::** **th** **ird one (0.4) the new one (0.3) is::**
152		**.hh[hh catching my eye=but then again you don't kno::w**
153	Neu:	[((moves head from side to side in a gesture that
154		seems to suggest equivocation))
155	Neu:	.tc[h
156	Pat:	**[‘cause it‐ be** **cause** **it's a new one.**
157	Neu:	I've a bit less experie‐ er‐ w(hh[hh. we(heh)ll (0.1)
158	Pat:	[Yea:h.
159	Neu:	£I've got a l(h)ot less exp(h)eri(h)ence (0.1) with
160		tha:t.£ .hhh
(Neurologist explains that he is happy to try this drug but would usually suggest it when other options were “closed off”).		
179	Neu:	I[::
180	Pat:	**[So really it's the first one that we’::re (0.1)**
181	Neu:	I‐ I think it's the first one=and it's not a perfect
182		drug.
183		(0.1)
184	Neu:	.hh u:m but for‐ e‐ sorry (0.1) not a perfect drug for
185		everyone.
186	Pat:	Oka(h)(h)y.

The components combine to offer the patient an explicit choice. In component 1, the neurologist names the information to come as a listing of ‘possibilities’ rather than a recommendation. In clearing space to ‘talk through these’ (line 2), he signals that the information is to be understood as part of a deliberation process (in contrast to prospective accounting for a decision already reached, as shown in Hudak *et al*. 2011). In component 2, the neurologist describes the options and their advantages and disadvantages, working hard to present each as reasonable for this patient, thus further constructing a choice. Finally, component 3 creates a slot for the patient to voice his views, with the potential to make the choice himself. As we have shown previously, this contrasts with making a recommendation in that ‘recommending turns do not invite patients to do more than simply accept the doctor's proposal’ (Toerien *et al*. 2013: 881). When responding to component 3, simple acceptance is inadequate. Instead, the decision‐making is placed in the patient's domain.

Thus, option‐listing can, demonstrably, be used to offer patients choice. This analysis accords with the self‐report finding (discussed above) that neurologists and patients almost always agreed that a choice had been offered following consultations in which option‐listing was used. Crucially, however, patients did not always take up this opportunity to choose. Extract 2 provides a contrast to Extract 1. Both patients are young men, accompanied by their parents. Both have epilepsy and are offered – by the same neurologist – a choice of anti‐epileptic drugs as a replacement for one that has not been working well. In both, the neurologist uses option‐listing in a similar way, announcing a choice to be made, listing more than one option, and seeking the patient's views on/selection from the list. Extract 2 shows this final component (boldfaced lines 108 and 110) and the responses produced by patient and parents.

Extract 2

**Table:** G10305: Epilepsy

(Previous lines entail presenting two options)		
108	Neu:	**.thhh Either >one of them,< (0.3) grab you?**
109		(0.6)
110	Neu:	**Any:** **p** **articular preference, (0.5) one or other,**
111		(1.1)
112	(Pat):	hhh[hhhh. ((sounds like breathy form of laughter))
113	Neu:	[It's difficult ‘cos they both work in relatively
114		similar ways:.
115		(0.1)
116	Mum:	I'm not [sure about the side effe:cts, but (.) has any of
117	(Pat):	[(Um:)
118	Mum:	them got (0.3) no(se) weight lo:ss ‘cos he's (0.1)
119		(kinda) [(a(h)wfully) thi(h)n [(a(h)s it [is
120	(Pat)/(Dad):	[No(h)
121	Neu:	[Right [No
122	Mum:	without losing any more wei(h)ght heh heh
(Neurologist explains that he wouldn't expect either drug to reduce appetite)		
133	Mum:	Right.
134		(0.6)
135	Dad:	(Depends on you::/you're::) (as: s:) (know) saying (1.1)
136		(professionally) what you think,

The contrast in responses to the option‐listing shown in Extracts 1 and 2 is marked. In 1, the patient takes up the opportunity to choose. Across lines 117–118, 151–152, and 180, we see him mirroring the neurologist's list by considering each option himself, finally concluding that the first is the one to try (line 180). This decision‐making is clearly guided by the neurologist, as he humorously recognises (lines 132, 134). Indeed, the patient's selection is built as a logical conclusion of the line of reasoning that has occurred, rather than simply as his personal preference: ‘so really it's the first one …’ (line 180). Nevertheless, it is the patient who articulates that conclusion, treating it as based on his evaluation of the evidence, his concerns about potential side effects, and the risks of trying a new drug.

By contrast, in Extract 2, the patient has difficulty in responding, evident in the silence at lines 109 and 111, and breathy laughter at 112. Subsequently, his mother asks a question (lines 116–122) and his father seeks the neurologist's view (lines 135–136). This results in a recommendation, which the family accepts, thus making this a matter of ‘assent’, rather than an expression of an independent ‘choice’ (Pilnick, 2008). Nevertheless, the family's responses indicate an understanding that the patient has been invited to choose. First, the patient's difficulty in responding is treated by the neurologist as a difficulty of choosing (lines 113–114). Second, when the patient's mother steps in, she orients to a possible basis for choosing that has not been explored (a possible side effect). Finally, the father's response counters the action of the neurologist's patient view elicitor (lines 108 and 110) with a request for his professional opinion, displaying an understanding that what has unfolded is not hearable as a recommendation. In resisting making a choice, he is recognising that a choice has been offered (see Reuber *et al*. 2015).

These contrasting cases demonstrate – in practice – the distinction between having a choice and making one. Following both consultations, patient and neurologist reported that a choice had been offered. Our data indicate, then, that option‐listing does not lose its capacity to generate the perception of choice even if the neurologist makes the decision.

### A hybrid practice: replacing the PVE with a recommendation

Extract 3 shows how option‐listing can be adapted (see also Reuber *et al*. 2015). The neurologist produces the first two components (see boldfaced lines 9–15). Thereafter, however, he deviates from the pattern shown above. In place of a patient view elicitor (component 3) – which could have been produced at lines 16–18 – he makes a recommendation for the second option (lines 17–18). Thus, rather than opening up space for the patient to make a selection, the neurologist transforms the list into a proposal, to be accepted or resisted. Moreover, he moves quickly (line 18) into further information provision (projected by a marked in‐breath), thereby leaving no room for a verbal response.

Extract 3

**Table:** G09701: diagnosis unknown; possibly small vessel cerebrovascular disease

01		Neu:	No:w .hh the difficulty is you had this test done for
02			something else and it's turned up (#another#) you know an
03			unexpected [finding, [so we’:re left with an abnormal
04		Pat:	[Yeah [yeah.
05		Neu:	test result whereas you don't have any kind of
06			disease .hhh symptoms to go with [that.
07		Pat:	[Mhm
08			(0.4)
09		Neu:	**U::m (1.2) .t (and) there're a couple of options he:re.**
10			**(0.2) .hhh We could jus:t (0.3) not do anything, [hh.**
11		Pat:	[Mhm
12		Neu:	**E::r and say “these are=incidental findings=not let‐ (.)**
13			**let's not worry about them,” .hhhh We could take it a**
14			**little bit further and‐ and investiga:te, (.) #for# (0.6)**
15			**possible causes of this:,**
16			(0.2)
17	➔	Neu:	U::m and (0.1) er that‐ that's: (0.2) prob’ly what I
18	➔		would recommend doing. .HHHhh No::w as I said (0.3)
19			these look (.) like (0.6) areas where the blood supply to
20			the brain isn't as good as it should be, they don't look
21			like areas of inflammation.
22		Pat:	**Mhm**
23		Neu:	But .hhhh (0.3) really I think to investigate this fully
24			what I'd like to do is a number of blood tests:,
25		Pat:	**Mhm**
26		Neu:	.hh And also do a thing called a lumbar puncture.
27			(0.2)
28		Pat:	**Yeh**.
29		Neu:	U::m that involves taking some spinal fluid out of the
30			back under local anaesthetic=it's a very straight forward
31			proc[edure.
32		Pat:	[**Okay**
33		Neu:	.HHHHhh But it would allo:w us to (0.4) know whether
34			these are inflammatory or not.=As I said looking at the
35			scan they don't look like inflammation=
36		Pat:	=**Okay**
37		Neu:	But it's a way of being more su::re.=
38		Pat:	=**Ye[h.**
39		Neu:	[.hhhh A::nd the blood test (we l‐)/(really) that we
40			(.) would do would be to look for causes of (0.5) you
41			know premature what we call vascular disease or (0.2) you
42			know (0.2) conditions that can affect the blood vessels.
43			(0.1)
44		Neu:	.hh Um there are various (0.6) what we call (0.3)
45			vasculitides or vasculitis which can do tha:t but [.hhhhh
46		Pat:	[**Yeh**
47		Neu:	(I‐) that's what I would sug[gest we do=if everything
48		Pat:	[**>Okay<**
49			comes back normal (it's) reassuring
50		Neu:	w[e don't need to do anything fu::rther.
51		Pat:	[**Yeah**
52			(0.4)
53		Pat:	**(The) what kinda time factor is involved? (.) in these**
54			**test thing(s) (doctor)**

Responding to the neurologist's elaboration of, and account for, his recommendation, the patient produces minimal acknowledgements (lines 22, 25), several more overtly aligning acknowledgements (lines 28, 36, 38, 46 and 51), and some possible indicators of accepting the recommendation (lines 32 and 48; boldface used to highlight these responses across lines 22–51; Gardner 2001). He does not voice a view or treat himself as selecting from the list. When he produces a fuller turn (boldfaced lines 53–54) this is to ask about the timing of the tests. This foreshadows a question about his life insurance rather than any resistance. By replacing the third component of option‐listing with a recommendation, the neurologist significantly alters the way in which the option‐listing functions – from creating a moment of choice for the patient, to proposing that a (professional) conclusion about what is best has already been reached. This is reflected in the patient's (accepting) response. It is striking, therefore, that even in this case, neurologist and patient agreed that choice was offered – which was true also of other cases in which a recommendation was made in conjunction with option‐listing (see Extract 16 in Reuber *et al*. 2015).

### Using option‐listing to pursue acceptance of a recommendation

In Extract 3, the neurologist's preference was somewhat indexed in how he presented the options: the minimising ‘just’ at line 10, and the implied risk of missing a serious disease make it hearable, before the explicit recommendation, that he is likely to endorse further testing. Nevertheless, he provides some basis for choosing to do nothing: the patient has no symptoms and these findings arose by chance when testing for something else (lines 1–6). Moreover, he makes no overt recommendation prior to the option‐listing. Extracts 4a–b, by contrast, show a decision sequence that begins with a recommendation (see boldfaced lines 1 and 7–11), acceptance of which the neurologist pursues repeatedly, before using option‐listing (see Reuber *et al*. 2015). The recommendation is to monitor the patient's undiagnosed ‘turns’ (lines 1 and 7–11), conveyed as the neurologist's view (‘I think’, lines 1 and 7–8) on what is best. It is given extra weight through the imperative, ‘we have to’ (lines 1 and 8). The neurologist does not, at this stage, raise the option of doing nothing. Although his proposal to try home monitoring (lines 9–11) is mitigated by the ‘I wonder if’ construction (Curl and Drew 2008), the question, for him, is clearly how best to monitor (not whether to).

Extract 4a

**Table:** G00604: Could be epilepsy or non‐epileptic seizures

01	Neu:	>.hh< Right. **I think what we have to do he:re. is** (1.0)
02		e:r how‐ how long were you in ((centre name^1^)) before,
03	Mum:	A mont[h.
04	Pat:	[A month.=
05	Neu:	=A month alright.
06	Pat:	[Yea::h.
[((Patient is focused on her seizure diary and appears to be about to start another turn when Neurologist starts speaking))		
07	Neu:	Well (.) I hesitate to do that again but (.) **I think**
08		**(0.2) I mean‐ I mean I think we have to try and record**
09		**some of these turns. .hh And I wonder if the best way to**
10		**do it would be to give you a wee monitor to go ↑home**
11		**with,**
12		[(0.8)
[((Patient moves towards Neurologist, holding out diary))		
13	Neu:	**What do yo[u think of that.**
14	Pat:	[(Basically)
15		(0.4)
16	Pat:	(wa::y) is (happening is) .hh I have a few days o:[:ff.
((During lines 14‐16, Patient shows Neurologist her record of her ‘turns’))		
17	Neu:	[Aye.
18		(0.5)
19	Pat:	Few days o:n.
20	Neu:	Right.

The patient has a mild learning difficulty, which may partially account for how she and the neurologist pursue parallel agendas. Here, she is consulting her seizure diary, used earlier to try to establish how long she can go without a ‘turn’. She pursues this as the neurologist tries to move on to what to do next (lines 6–13). In the absence of a fitted response to his recommendation (which could have occurred at line 12), the neurologist seeks the patient's view (boldface line 13, showing his pursuit). Her response remains rooted in how often she experiences her ‘turns’ (lines 14–16 and 19). This misalignment continues in a manner that could be understood as resistance to monitoring (data not shown). What matters, for our purposes, is that the neurologist employs option‐listing only after he has failed to secure the patient's acceptance of his recommendation.

We rejoin the interaction at a point where the neurologist is trying (again) to gain this acceptance (Extract 4b, lines 57–63). He looks set to reissue his recommendation (‘y‐ you could’, line 57) but instead provides a justification, strongly positioning the recommended investigation as a necessary precursor to treatment (lines 57–63). He then employs a condensed but clear version of option‐listing (see boldfaced lines, 65–70). At lines 65–66, he flags up that there is a decision to be made, placing this in the patient's domain: ‘you need to decide what you would like us to do’ (component 1). He then lists two options, formatted as an interrogative (lines 66–70), thereby folding the action of listing (component 2) and seeking the patient's view (component 3) into one unit.

Extract 4b

**Table:** G00604

57	Neu:	E:r (0.4) y‐ you could ((coughs)) (.) ‘cos: at the end of
58		the da::y (0.2) basically if we can record these turns we
59		ma:y be able to help you, (.) but if we ca:n't record
60		these turns (0.2) we're probably not going to be able to
61		help you, (0.4) So: ((clears throat)) and‐ and we
62		wouldn't be bringing you back and forth to the clinic if
63		we can't help you obviously.
64		(.)
65	Neu:	**.hh So: what we need to do:: er clearly is you need to**
66		**deci:de (.) what‐ what you would like us to do:=d'you**
67		**want us to keep trying to (0.2) record them so we're**
68		**(0.2) com** **ple** **tely sure what everything is and we can (0.2)**
69		**maybe** **treat** **↑them, (0.4) or d'you want to: (0.2) kind of**
70		**(1.0) just soldier on as you a:re.**
71		(.)
72	Neu:	(you [‘cos)
73	Mum:	[No because it's kind of uh (0.5) (it take‐)
74		(containing) me to whe::re .hh what I can do::,
75	Neu:	Mhm.
(Neurologist pursues Patient's view, they resolve a minor misunderstanding, and she indicates that she does not want to ‘soldier on’)		
103	Neu:	Yeah (okay). Well (.) d'you want us to (.) do some
104		recordings then to see what we [can find out
105	Mum:	[Yea::h.
106	Pat:	See what you can do(h.),
107	Neu:	Yeah okay, I:’ll arrange that then.

The design of the options is clearly not neutral. In constructing the option of monitoring, the neurologist not only upgrades its utility (line 68), but makes treatment contingent on this investigation (lines 68–69). Moreover, the alternative is set up as unsatisfactory for the patient: having to ‘soldier on’ entails coping in the face of adversity, which could be lessened if they identified an appropriate treatment. Moreover, this amounts to ‘doing nothing’, which could be argued to construct little choice. The terms of the decision are set, then, to make it difficult to bid for no monitoring. Although the neurologist has used the machinery of option‐listing, he has done so in a way that constructs another version of his recommendation, pursuing acceptance thereof.

Mum addresses the second option as if it were a single yes/no question,^1^ rejecting it (line 73) and accounting for this in terms of her concerns (lines 73–74). The upfront ‘no’ suggests that this is produced as an alignment with the neurologist's position: that continuing as they are is not the best option (see Pomerantz and Heritage 2013). Thus, she displays an understanding that the two options are not equally weighted. The neurologist then seeks the patient's agreement, using another PVE, the eventual upshot of which is an agreement by the patient that she also wants to find a solution. The neurologist finally secures a clear agreement from Mum (line 105) and patient to undertake testing (line 106).

Comparing Extracts 1 and 4a–b, we see the malleability of option‐listing. Although both produce a list from which the patient may choose, the neurologist in 1 constructs viable alternatives, whereas the neurologist in 4b uses option‐listing to pursue acceptance of his recommended option. Nevertheless, in their post‐consultation questionnaires, participants in both reported that a choice had been offered.^2^ Thus, even when option‐listing was used in a way that seems to stretch the limits of ‘choice’ close to breaking point, it was reportedly perceived as making choice available.

### A deviant case

Extract 5 shows a deviant case in that the neurologist reported that a choice had been offered, and the patient that it had not. The patient has long‐standing epilepsy, which is not fully controlled. The neurologist has concluded that they should reduce one of her drugs because it appears to be causing fatigue and not improving seizure control. The question is what else they might try. The neurologist makes a bad news announcement: they have, more or less, run out of alternatives and ‘the chances of getting [her] seizure free are really very low indeed’. Implicitly, he is recommending continuing with her current medication. In this regard, his turn is comparable to cases in our no‐choice subset (i.e. subset 4, Table 1), which typically involved recommendations for doing nothing or nothing new (Reuber *et al*. 2015).

Against this backdrop, the neurologist uses option‐listing (see boldface text across lines 15–39). Again, we see the familiar three components. He flags up that a decision is to be made (line 15), lists two alternatives (lines 15–16 and 18–22), places the decision in the patient's domain (lines 15 and 23–24), and uses a PVE to hand the decision to the patient (lines 38–39). When this fails to elicit a response (line 40), the neurologist returns to his original position: that he has little to offer.

Extract 5

**Table:** G08904

(Previous lines entail flagging up that they don't have many new options to offer this patient)		
15	Neu:	E:r (0.5) **and it's up to you a bit, u::m .hh some people**
16		**say “well look I need to try every drug,”**
17		(0.5)
18	Neu:	**Er (I‐) you know (0.1) whatev[e:r, e:r** **oth** **er (0.1)**
19	(Hus):	[Mm
20	Neu:	**patients say “we:ll (0.3) you kno::w, (0.3) #I:: # I've**
21		**been through all this befo:re (0.1) side effects upse:t**
22		**(.) all that sort of thing .hhh I'll stick where I ↑a:m”**
23		**.hhh And it depends a wee bit on you: (0.3) you know what**
24		**your attitude is.** .hhh There're certainly a couple of
25		medications there we could try::, What are the chances
26		they'll work? (0.3) Quite low.
(Neurologist specifies that the chances are “of the order of a couple of percent”)		
38	Neu:	But not zero, (0.1) **so:: it's really up to you whether you**
39		**want to try them or not.**
40		(0.8) ((Pat looks at neu))
		(Neurologist further explicates the low chance of achieving full seizure freedom)
92	Neu:	(But) I‐ I‐ I mean if you do want to try them .hh you
93		know I have to be realistic with you and sa:y, you know
94		(0.2) the‐ the chances of them actually helping you out
95		are‐ are really pretty low.
96		(0.2)
97	Pat:	Yea:h.
98	Neu:	U:m .hh er just (0.4) you know and there is a downside to
99		trying new tablets.=So [it's‐ it's it's your choice.
100	Hus:	[Mm::: yea:h.
101		(1.3)
102	Pat:	.hh If that's the case then I'm as we[ll just
103	Hus:	[(You've) just gotta
104		live with the:
105	Neu:	M[m
106	Hus:	[situation you've got.
107		(0.4)
108	Neu:	.h[hh
109	Pat:	[I just need to st[ick with the si[tuation I've
110	Neu:	[°Yeah.° [°Yeah.°
111	Pat:	[got [(aye)
112	Hus:	[Mhm
113	Neu:	[°Okay°

The disagreement in the neurologist's and patient's self‐report data is evident *within* the consultation. The neurologist frames the decision as the patient's ‘choice’ (line 99), but the patient and her husband construct it starkly as a non‐choice, using the imperatives: ‘gotta live with [it]’ (lines 103–104) and ‘need to stick with the situation’ (line 109). The use of option‐listing – and even the phrase, ‘it's your choice’ – does not, then, guarantee that patients will experience themselves as having genuine alternative options.

However, this was the only case (for which we have valid data) where option‐listing was used to generate a decision about a test, treatment or referral that was not perceived by the patient as offering choice. What distinguishes this from the rest? Its unique feature lies in what it lacks: the construction of any option as likely to address the patient's trouble. Choice is there in the formal sense: the patient is, as the neurologist reports, given the option to try an alternative drug. However, choice is not there, as the patient reports, in a substantive sense: no option is presented as likely to make any difference (see Reuber *et al*. 2015).

## Discussion

Based on 223 neurology consultations and participants’ self‐reports following these, we have demonstrated that choice can be hearably offered through option‐listing. Our evidence takes two forms: first, option‐listing was the only practice identified as characteristic of those consultations in which neurologists and patients agreed – in their self‐reports – that choice had been offered. Second, analysis of the practice of option‐listing shows how it can be used to create a moment of choice for the patient in the consultation. However, we found that even when the patient resisted making the choice or the neurologist adapted the practice of option‐listing in significant ways, participants still agreed that a choice was offered. Our deviant case – in which the neurologist said a choice was offered and the patient said not – differed in one important respect: no option was constructed as likely to benefit the patient. This sheds light on the limits of option‐listing as a means of giving patients (the perception of) choice.

Previous research has shown that patients report concern about choice performed as ‘a politically driven box‐ticking exercise’ (Barnett *et al*. 2008: 611) and that clinicians can struggle to construct opportunities for independent choice (Pilnick 2008). One might, then, expect higher levels of disagreement about whether choice was offered, given that option‐listing was used in ways that stretched the limits of what could be analysed as ‘choice’. What are we to make of this? It seems reasonable to consider option‐listing to be a canonical method for offering choice. The menu is a ubiquitous example of option‐listing in everyday service encounters, and versions of option‐listing have been identified in offers of choice by carers to people with learning difficulties (Antaki *et al*. 2008). Our findings may, therefore, reflect a mundane understanding that menus give choice – even if highly constrained. Patients may also understand option‐listing as a practice for foregrounding their right to choose, even when the alternatives are not evenly balanced (either ‘in reality’ – however we may judge that – or in how the options are presented). Thus, patients may, justifiably, hear themselves as having been offered a choice even if only one option was presented as a viable solution. If so, there is a clear message for practitioners: option‐listing is a robust practice for generating the perception of choice, provided at least one option offers potential benefit. Whether clinicians should be generating such a perception (or under what circumstances) is, however, less clear.

There are three main reasons for caution. First, there is unresolved debate around whether having choice is necessarily better for patients. Psychological research, indicating positive consequences of choice, has been invoked to support the patient choice agenda. For example, studies have shown increased ‘intrinsic motivation, task performance, life skills, and higher outcome evaluations’ (Ogden *et al*. 2008: 614), which, intriguingly, have been found ‘to occur regardless of whether choice is actual, trivial, or illusory’ (Ogden *et al*. 2008: 614). Moreover, Ogden *et al*. showed that most participants rated having choice as beneficial. However, some research has indicated that – at least following extensive rather than limited choices – participants’ levels of motivation and satisfaction were lower (in Ogden *et al*. 2008). Ogden *et al*. also showed mixed responses when it came to making choices, with around two‐thirds of the sample being unsure or negative about this. Likewise, the consultants in Ford *et al*.'s (2003: 597) study were generally critical of expecting patients to select from among options, believing that they ‘should advise patients on the best course of action for them’. This chimes both with patients’ reports that choice could be experienced as a threat to their ‘faith in the expertise of the doctor at a time when they were most reliant upon it’ (Barnett *et al*. 2008: 612), and with our finding *that* some patients resisted making a choice and *how* they did so; e.g. the patient's father in Extract 2 sought the neurologist's professional opinion.^3^ Moreover, even if there are benefits from having choice, it is unclear whether these extend to all patient groups equally (Barnett *et al*. 2008).

A second reason for caution lies in how choice is offered. Based on our pilot work, we argued that, compared with recommending, option‐listing works to reduce the exercise of medical authority by placing the decision explicitly in the patient's domain and avoiding the need to ‘work with (or against) an expert opinion of what is best’ (Toerien *et al*. 2013: 886). As we demonstrate here, option‐listing can function in this way. But it need not. If used as shown in Extract 4b, option‐listing may function to pursue acceptance of the neurologist's preferred option. Antaki and Kent (2015: 29) have identified a similar practice in adult‐child interactions; for example, ‘would you put them neatly in the corner for mummy please or do you wanna go to bed’. They argue that, by offering alternatives entailing ‘a choice between two manifestly differently valenced courses of action’, adults are issuing directives, with an accompanying threat of what will happen if these are not followed. Similarly, in Extract 4b, having to ‘soldier on’ with no help from the clinic is the threatened negative consequence of failure to undergo monitoring. As Antaki and Kent (2015: 37) conclude: ‘the interactional effect is that [such turns] seemingly preserve the child's choice and agency, while tipping the balance heavily in favour of the adult's preference’.

Thus, if patients perceive option‐listing as offering choice even when it is functioning to seek their acceptance of a recommendation (particularly in the face of resistance), then option‐listing may sometimes operate less as a practice for reducing authority and more as one for disguising its exercise. Although there can be good reasons for persuading patients to do things they would rather not, such practices sit uncomfortably alongside the widespread endorsement of a more egalitarian relationship between doctors and patients. Simply advocating the use of option‐listing carries no guarantee that it will produce a genuine moment of choice for the patient in practice.

However, and this is the third reason for caution, it may be that the policy directive to increase ‘patient choice’ is a red herring. There is now a vast body of literature advocating a multi‐faceted model of ‘shared decision‐making’ (SDM), which allows for significant patient involvement but also pushes back against a consumerist model, in which the patient is left to choose as if making a purely preference‐based decision, like whether to order coffee or tea. SDM emphasises interactional approaches that facilitate informed decision‐making, which balances the patient's preferences and the current state of medical evidence (e.g. Elwyn and Edwards 2009). However, while SDM is widely accepted as an ideal, the policy literature slides between this broader concept and more specific calls for increased ‘patient choice’. Analysts and practitioners alike, then, need to be vigilant about what version of patient involvement is (often implicitly) being articulated.

To our knowledge, ours was the first study to focus on what constitutes, specifically, ‘patient choice’ from the dual perspective of participants’ self‐reports and close analysis of real decision‐making. Further research is needed to address the limitations of our study, which include the possibility that our findings could be specific to neurology, to secondary care, to NHS practice, and/or to chronic disorders. Future research might also seek more nuanced accounts of participants’ perceptions of choice using interviews instead of brief questionnaires.

This article has focused on the only practice for giving patients choice that we identified as characteristic of those consultations in which participants agreed that a choice had been offered. However, option‐listing was not the only practice used to initiate decision‐making in this subset. Moreover, in roughly a third of our consultations neurologists and patients disagreed about whether choice had been offered. It is clear, then, that what constitutes patient choice is far from self‐evident. Given the extent to which NHS policy encourages clinicians to offer patients more choice, unpicking what this means in practice should be a priority.

## Notes

1 It's worth noting that one would expect the second option to be addressed first, as per Sacks's (1987) findings on the preference for ‘contiguity in sequences in conversation’. This may also contribute to the upfront production of ‘no’ (with thanks to an anonymous reviewer for this observation).

2 While we cannot rule out the possibility that the patient in Extracts 4a–b may have had limited understanding of the post‐consultation questionnaire (given her mild learning difficulty), the questionnaires were completed with our research assistant and the patient's mum present to provide help. Moreover, there is evidence of sufficient understanding in the patient's other answers for us to consider this to be a valid representation of her perception of choice.

3 With thanks to an anonymous reviewer for this observation.

## References

[shil12766-bib-0001] Antaki, C. , Finlay, W. , Walton, C. and Pate, L. (2008) Offering choices to people with intellectual disabilities: An interactional study, Journal of Intellectual Disability Research, 52, 12, 1165–75. 18627430 10.1111/j.1365-2788.2008.01101.x

[shil12766-bib-0002] Barnett, J. , Ogden, J. and Daniells, E. (2008) The value of choice: A qualitative study, British Journal of General Practice, 58, 554, 609–13. 18801277 10.3399/bjgp08X330717 PMC2529197

[shil12766-bib-0003] Costa‐Font, J. and Zigante, V. (2016) The choice agenda in European health systems: The role of middle‐class demands, Public Money & Management, 36, 6, 409–16.

[shil12766-bib-0004] Curl, T. and Drew, P. (2008) Contingency and action: A comparison of two forms of requesting, Research on Language & Social Interaction, 41, 2, 129–53.

[shil12766-bib-0005] Dent, M. (2006) Patient choice and medicine in health care: Responsibilization, governance and proto‐professionalization, Public Management Review, 8, 3, 449–62.

[shil12766-bib-0006] Department of Health (2005) The National Service Framework for Long‐term Conditions. London: Department of Health.

[shil12766-bib-0007] Department of Health (2007) Choice Matters: 2007–08 Putting Patients in Control. London: Department of Health.

[shil12766-bib-0008] Department of Health (2013) NHS 2013/14 Choice Framework. London: Department of Health.

[shil12766-bib-0009] Elwyn, G. and Edwards, A. (2009) Shared Decision‐making in Health Care: Achieving Evidence‐based Patient Choice, 2nd edn Oxford: Oxford University Press.

[shil12766-bib-0010] Ford, S. , Schofield, T. and Hope, T. (2003) What are the ingredients for a successful evidence‐based patient choice consultation? A qualitative study, Social Science & Medicine, 56, 3, 589–602. 12570976 10.1016/s0277-9536(02)00056-4

[shil12766-bib-0501] Gardner, R. (2001) When listeners talk: Response tokens and listener stance. Amsterdam/Philadelphia: John Benjamins Publishing Company.

[shil12766-bib-0011] Heritage J. and Maynard D.W. (eds) (2006) Communication in Medical Care: Interaction between Primary Care Physicians and Patients. Cambridge: Cambridge University Press.

[shil12766-bib-0502] Hudak, P.L. , Clark, S.J. and Raymond, G. (2011) How surgeons design treatment recommendations in orthopaedic surgery, Social Science & Medicine, 73, 7, 1028–36. 21855192 10.1016/j.socscimed.2011.06.061

[shil12766-bib-0012] McCorry, D. , Marson, T. and Jacoby, A. (2004) Understanding routine antiepileptic drug decisions: A qualitative analysis of patients’ accounts of hospital consultations, Epilepsy & Behavior, 14, 1, 210–4. 10.1016/j.yebeh.2008.10.010 18977312

[shil12766-bib-0013] Ogden, J. , Daniells, E. and Barnett, J. (2008) The value of choice: Development of a new measurement tool, British Journal of General Practice, 58, 554, 614–8. 18801278 10.3399/bjgp08X330735 PMC2529198

[shil12766-bib-0014] Pietrolongo, E. , Giordano, A. , Kleinefeld, M. , Confalonieri, P. , *et al* (2013) Decision‐making in Multiple Sclerosis consultations in Italy: Third observer and patient assessments, PLoS ONE, 8, 4, e60721. 23565270 10.1371/journal.pone.0060721 PMC3614559

[shil12766-bib-0015] Pilnick, A. (2008) ‘It's something for you both to think about’: Choice and decision making in nuchal translucency screening for Down's syndrome, Sociology of Health & Illness, 30, 4, 511–30. 18298631 10.1111/j.1467-9566.2007.01071.x

[shil12766-bib-0016] Pomerantz, A. and Heritage, J. (2013) Preference In Sidnell J. and Stivers T. (eds) Handbook of Conversation Analysis. Oxford: Wiley‐Blackwell.

[shil12766-bib-0017] Reuber, M. , Toerien, M. , Shaw, R. and Duncan, R. (2015) Delivering patient choice in clinical practice: A conversation analytic study of communication practices used in neurology clinics to involve patients in decision‐making. Southampton: NIHR Journals Library Available from https://www.ncbi.nlm.nih.gov/books/NBK279940/ (Last accessed on 31 July 2018). 25834883

[shil12766-bib-0018] Sacks, H. (1987) On the preferences for agreement and contiguity in sequences in conversation In Button G. and Lee J.R.E. (eds) Talk and Social Organisation. Clevedon: Multilingual Matters.

[shil12766-bib-0019] Toerien, M. , Shaw, R. and Reuber, M. (2013) Initiating decision‐making in neurology consultations: ‘recommending’ versus ‘option‐listing’ and the implications for medical authority, Sociology of Health and Illness, 35, 6, 873–90. 23550963 10.1111/1467-9566.12000

[shil12766-bib-0020] Wiseman, H. , Chappell, P. , Toerien, M. , Shaw, R. , *et al* (2016) Do patients want choice? An observational study of neurology consultations, Patient Education & Counseling, 99, 7, 1170–8. 26961278 10.1016/j.pec.2016.02.015

